# How is the Lives Saved Tool (LiST) used in the global health community? Results of a mixed-methods LiST user study

**DOI:** 10.1186/s12889-017-4750-5

**Published:** 2017-11-07

**Authors:** Angela R. Stegmuller, Andrew Self, Kate Litvin, Timothy Roberton

**Affiliations:** Department of International Health, Johns Hopkins Bloomberg School of Public Health, Baltimore, MD USA

**Keywords:** Lives saved tool, LiST, Spectrum, Avenir health

## Abstract

**Background:**

The Lives Saved Tool (LiST) is a computer-based model that estimates the impact of scaling up key interventions to improve maternal, newborn and child health. Initially developed to inform the *Lancet* Child Survival Series of 2003, the functionality and scope of LiST have been expanded greatly over the past 10 years. This study sought to “take stock” of how LiST is now being used and for what purposes.

**Methods:**

We conducted a quantitative survey of LiST users, qualitative interviews with a smaller sample of LiST users and members of the LiST team at Johns Hopkins University, and a literature review of studies involving LiST analyses.

**Results:**

LiST is being used by donors, international organizations, governments, NGOs and academic institutions to assist program evaluation, inform strategic planning and evidenced-based decision-making, and advocate for high-impact interventions. Some organizations have integrated LiST into internal workflows and built in-house capacity for using LiST, while other organizations rely on the LiST team for support and to outsource analyses. In addition to being a popular stand-alone software, LiST is used as a calculation engine for other applications.

**Conclusions:**

The Lives Saved Tool has been reported to be a useful model in maternal, newborn, and child health. With continued commitment, LiST should remain as a part of the international health toolkit used to assess maternal, newborn and child health programs.

**Electronic supplementary material:**

The online version of this article (10.1186/s12889-017-4750-5) contains supplementary material, which is available to authorized users.

## Background

The Lives Saved Tool (LiST) is based on a modeling approach developed over ten years ago to estimate the impact of scaling up community-based interventions on child mortality for the 2003 *Lancet* Child Survival Series [1, 2]. Over time, LiST evolved to include facility-based interventions that impact newborn mortality [3, 4]. Later, stunting and wasting risk factors were added to the model for the 2008 *Lancet* Nutrition Series [5]. With financial support from the Bill & Melinda Gates Foundation, the model was integrated into Spectrum, an analytical health program planning tool [6]. In 2011, interventions that impact stillbirths, birth outcomes and maternal mortality [7] were added to the model, in addition to diarrhea and pneumonia incidence in 2013 [8].

The integration of LiST into Spectrum allowed the implementation of demography, family planning and HIV/AIDS interventions into LiST projections via the Demographic Projection (DemProj), AIDS Impact (AIM) and Family Planning (FamPlan) modules [9]. Additionally, LiST’s ability to estimate lives saved through multiple, age-specific interventions at a time gives users the ability to more accurately model the country-context. LiST’s method of first estimating impact of prevention interventions followed by treatment interventions eliminates “double counting” errors [10]. Lastly, the body of evidence behind LiST is extensive and continues to be updated. The Child Health Epidemiology Reference Group (CHERG) previously reviewed the best available evidence of the effectiveness of interventions in the model [11, 12]. Currently, the LiST team commissions outside experts to review the model’s assumptions and strengthen the evidence-base. Without this process, the Lives Saved Tool would lack scientific integrity.

Until now, discussion in the literature of how LiST has been used by public health practitioners has been limited. Few users report back to the LiST team on how they have used the model. The purpose of this paper is to document the ways LiST is used. We report here on who LiST users are, the various applications of the model, organizations’ institutional capacity for using LiST and the various tools that have incorporated LiST as a calculation engine within other models or applications.

## Methods

This study used three methods of data collection: (1) a quantitative survey of LiST users; (2) interviews with a smaller sample of LiST users and members of the LiST team at Johns Hopkins University; and (3) a literature review of studies involving LiST analyses. Each of these activities were undertaken by members of the LiST team, as part of a broader strategy to understand the ways in which LiST is being used and to identify priorities for future LiST development. We analyzed data from all three methods concurrently to develop findings on how LiST is being used in the global health community.

The quantitative survey consisted of a structured, self-administered questionnaire distributed via Google Forms. We invited all subscribers to the LiST electronic mailing list to take the survey. At the time of the invitation, the list had approximately 1550 subscribers, of whom 106 (6.8%) responded. This low response rate was likely due to the fact that many people on the mailing list were not active users of LiST or entered email addresses that were no longer monitored. An email was sent to subscribers inviting them to complete the questionnaire, with a follow-up email sent three weeks later. Subscribers to the mailing list consist of NGO staff, consultants, academics, government staff and donor representatives. The majority of respondents who reported their affiliation were from academia and NGOs (31.4% and 25.6% respectively). Questions in the online questionnaire were predominantly multiple-choice, focusing on people’s backgrounds, experiences using LiST and their sources for LiST training and support; for example, “How often do you use LiST for analysis?”, “For which of the following purposes do you use LiST?”, and “Where do you get information about how to use LiST?”. A copy of the questionnaire is included as Additional file 1.

In addition to the quantitative survey, we undertook qualitative interviews with a subset of users. Two interviewers, graduate students who completed coursework in public health research methods, used a semi-structured guide to conduct interviews in-person and by phone. We used purposive sampling to select respondents from the LiST mailing group with a range of backgrounds and organizations, including technical staff who conduct LiST analyses themselves, and policy and managerial staff who either use LiST themselves or commission LiST analyses from others. Twenty-six people were invited to participate in interviews, of whom 21 (81%) participated. Interview questions focused on respondents’ experiences using LiST, the nature of their work using the tool, data sources, countries of interest and other details related to the specific projects. Interviewers also met with members of the LiST team, asking them similar questions from their perspective of having supported LiST analyses for various organizations. Throughout data collection, interviewers met as a team with the study coordinator to review preliminary findings and identify themes for greater focus. These meetings also served to ensure consistency in questioning. All interviews were audio recorded. Interviewers took notes during interviews and used audio recordings to verify data and quotations. The survey is included in Additional file 2.

**Fig. 1:**
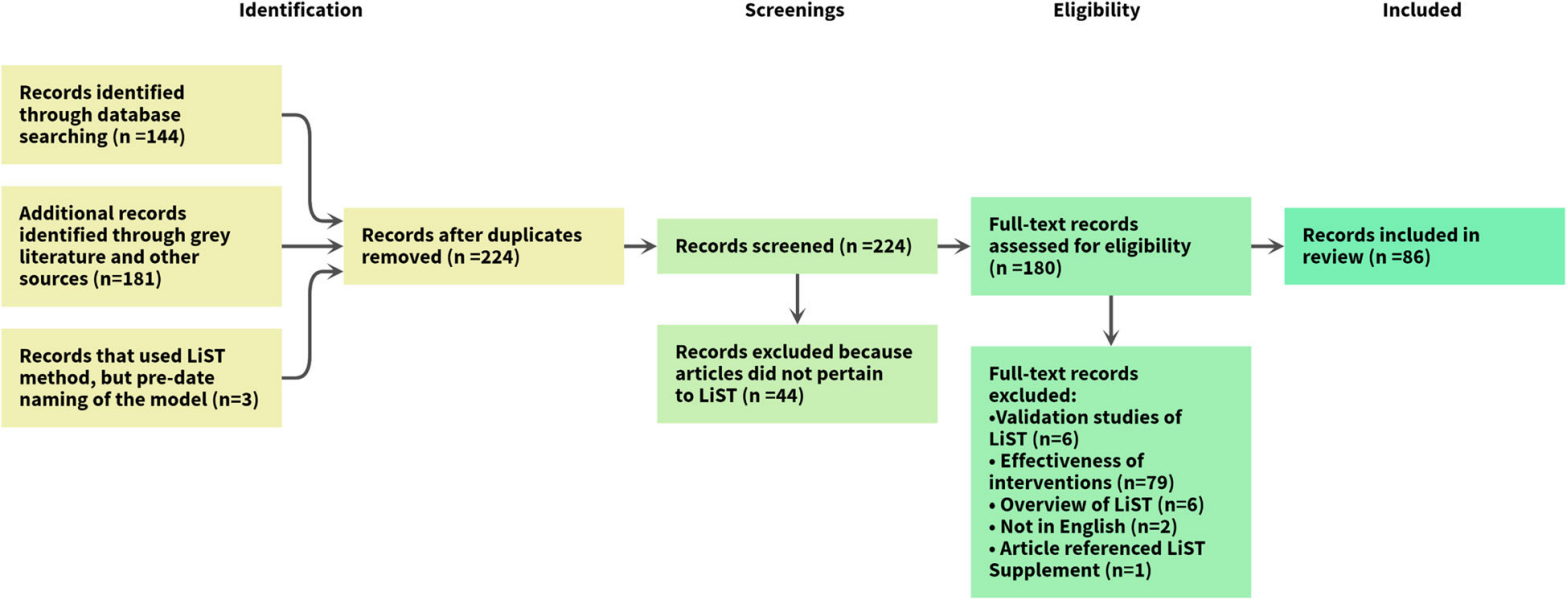
Literature review process

Finally, we conducted a literature review to understand how LiST is being applied and used for published analyses. We searched online databases of academic journals using the search terms “Lives Saved Tool,” “Lives Saved LiST” and “Lives Saved Spectrum” on PubMed. Articles that used LiST for application were included in this study, while studies commissioned to further the scientific basis of LiST itself were excluded. Grey literature was identified through the LiST website, web searches and personal communication with LiST team members. Only articles in English were included. Each article was reviewed and information was extracted on the organization conducting the study, the nature of the analysis and the type of program or policy in question. Figure 1 outlines the review process.

## Results

We present results on four topics: the people and organizations that are using LiST, the purposes for the LiST analyses that are conducted, organizations’ institutional capacity for LiST and other third-party models that build upon LiST.

### Who is using LiST, and how?

The results of our quantitative survey and literature review show that LiST has been used by a variety of actors across the maternal health, child health and nutrition communities. Stakeholders include donors and international organizations at the global level, national governments at country level, NGOs and implementing partners at a local level and academic users at all three levels. For examples of such LiST users, see Table 1.

**Table 1:** Examples of LiST users categorized by organization

Organization Type	Examples
International Donor	Bill & Melinda Gates Foundation, Children’s Investment Fund Foundation
International Organization	Gavi, the Vaccine Alliance, UNICEF, World Health Organization
Development and Aid Agency	Global Affairs Canada, UK Department for International Development, US Agency for International Development
Country Government	Democratic Republic of Congo, India, Malawi, Mali, Mozambique, Nigeria, Peru
Nongovernmental Organization	Catholic Relief Services, Jhpiego, Management Sciences for Health, PATH, Population Services International, Save the Children, World Vision International
Academic Institution	Aga Khan University, London School of Hygiene and Tropical Medicine, ​Johns Hopkins Bloomberg School of Public Health
Other	Independent consultants, independent research institutes

International donors, organizations, and aid agencies have used LiST for broad policy-setting, typically to assess the potential impact of different packages of interventions and to choose between them. Additionally, LiST is used to evaluate the impact that organizations’ programs have on mortality. The UK’s Department of International Development (DFID) recently outlined its evaluation strategy, which utilizes LiST to estimate the impact of DFID programs in conjunction with other existing programs [13]. Another example includes Gavi, the Vaccine Alliance, which has used LiST to assess the impact of its vaccine scale-up programs in priority countries as well as to inform policies, investments and strategies.

At a national level, government staff members have used LiST for analyzing trends in mortality within their country [14] and for prioritizing and planning health programs [15]. For example, the National Statistics Office of Malawi used LiST to determine the country’s progress in attaining the Millennium Development Goal (MDG) 4 target of reducing under-5 mortality by two-thirds between 1990 and 2015 [14]. In 2013, the Indian government used LiST as part of their strategic planning process for maternal and child health to determine key interventions to be scaled up [16].

At an even more localized level, LiST is used to assist in program planning and evaluation of NGO programs. For instance, a 2015 paper evaluated care group programs implemented by NGOs such as World Relief and Food for the Hungry in Mozambique, Rwanda, Cambodia, and Kenya [17]. Furthermore, some qualitative survey participants from NGOs reported that they incorporate LiST into their internal procedures for monitoring and evaluation.

### What is LiST used for?

In looking at our data we observed three broad purposes for which LiST has been used: evaluation, strategic planning and advocacy. We defined evaluation as retrospective analyses that determine changes in outcomes over time, including evaluations of government-led national plans to reduce mortality as well as evaluations of smaller-scale (e.g., NGO-led) health programs. Strategic planning included prospective analyses used to determine priorities for improving maternal, newborn and child health (MNCH), often involving costing estimates and program planning exercises. Lastly, we defined advocacy as a prospective analysis of lives saved. Such analyses set coverage to universal or aspirational targets with the intention of determining the maximum potential reduction in neonatal, under-five and/or maternal mortality.

#### Evaluation

According to our quantitative survey, the most widely reported use of LiST was program monitoring and evaluation. Users reported that LiST plays a vital role in evaluation, because when mortality data are not available, LiST allows users to estimate mortality impact based on coverage data. Modeling has been particularly useful in NGO program evaluations, where mortality is difficult to measure due the timing and costs of evaluation [18]. One qualitative survey respondent mentioned this:

*“LiST provides us with information for evaluation, lives saved, deaths averted for a 3-5 year health program. It is hard to have mortality data from this. It is impossible to measure for one intervention over such a short period. LiST helps give mortality data.”*

*-Monitoring and evaluation officer at an NGO*

In Uganda, LiST was used to evaluate the integrated community case management (iCCM) strategy [19]. The research team used household surveys conducted in both intervention and comparison areas to obtain coverage data for LiST. By entering these coverage data into LiST, the team was able to determine that under-five mortality fell in intervention areas. Furthermore, LiST has specifically been described as a helpful model to evaluate iCCM in complex country situations [20]. Due to LiST’s ability to attribute mortality reductions to specific interventions, the model has been useful when other programs are rolled out in addition to iCCM.

#### Strategic planning

A second way in which LiST is commonly used is for strategic planning purposes. The results given by LiST allow users to identify the impact of scaling up different packages of interventions, and thus which combination of interventions would most greatly reduce child or maternal mortality:

“*One of the things we also are challenged with regularly is how to make strategic decisions for design. When you have a context where there are so many issues, how to prioritize what to focus, you know kind of step by step, what is our highest priority and our secondary priorities. LiST can really help to make those decisions. It can help show empirically what is going to make the greatest impact on lives saved when otherwise it is quite hard to make those decisions.”*

*-Program coordinator at an NGO*

In 2007 and 2008, Ghana, Burkina Faso and Malawi sought funding to scale up 13 to 20 interventions that aimed to reduce under-five mortality by 20% by 2011. The feasibility of reaching coverage targets for such interventions concerned many stakeholders, so the country teams were encouraged to use LiST to more effectively prioritize interventions. Using LiST, the teams determined that only four to five high-impact interventions were needed to achieve a 20% reduction in under-five mortality [15].

In South Africa, LiST was used to determine priority interventions to reduce child, neonatal and maternal mortality in addition to stillbirths in the KwaZulu-Natal province [21]. In 2014, the Maternal, Child and Women’s Health Unit within the Department of Health began designing its strategic plan for 2015 to 2019. The department used LiST to identify a set of interventions to reduce maternal, newborn and child deaths and stillbirths in the province as well as the costs associated with scale-up. The analysis involved reviewing the default data in the LiST software for South Africa and updating coverage values to reflect the KwaZulu-Natal province. The team then created two scale-up scenarios: a “full coverage” scenario of 95% for all interventions and an “achievable coverage” scale-up based on target coverage levels determined by province experts. Additionally, the LiST Costing module was used to determine the cost-effectiveness of key interventions. The South African team revised the medical staff salary estimates in the software, but used the default costs for medicine and supplies. The team concluded that seventeen interventions plus family planning were both impactful and cost effective for averting deaths in the KwaZulu-Natal province [21].

LiST was also used for strategic planning for achievement of the MDGs as a part of the Countdown to 2015 project. In 2003, as an outgrowth of the *Lancet* Child Survival Series [22], academics, UN organizations and other partners collaborated to create the Countdown to 2015 to monitor and track progress toward the Millennium Development Goals for maternal and child health. This group combined information from various sources to create country profiles to track under-five mortality as well as changes in coverage of proven effective interventions for the major causes of under-five mortality. In addition to country profiles, the Countdown group published case study reports that focused on new issues and examples for countries. In these case studies, country teams compiled information related to policy, financing, coverage and other program variables to provide a better understanding on the factors related to progress in maternal, newborn and child health. The Lives Saved Tool played a central role in these case studies as the model allowed the user to attribute changes in mortality and nutritional status to specific interventions. Using the Malawi Country Case study as an example, the LiST analysis showed that under-five mortality fell in Malawi during the period of 2000 to 2013 [14]. The rapid decrease in under-five mortality was driven primarily by reductions in stunting and wasting, increased ownership of insecticide-treated nets, increased careseeking and treatment for diarrhea and pneumonia as well as the introduction and high coverage of *Haemophilus influenzae* type B and pneumococcal vaccines. This same strategy of using the Lives Saved Tool to help determine the causal relationships between program activities, coverage changes and mortality reduction was used in case studies for Peru, Afghanistan, Niger and Tanzania [23–26].

Lastly, LiST has aided MNCH experts in the preparation of proposals. Participants from the qualitative survey noted the importance of modeling during the proposal writing process, mentioning that donors prefer that implementers model the possible impact of their program.

#### Advocacy

Of the users surveyed in our quantitative study, 28% of respondents reported using LiST for advocacy purposes. Advocacy analyses sought to highlight the potential benefits of scaling-up of key health interventions, notably for pneumonia diarrhea, and malaria. For example, Johns Hopkins School of Public Health used LiST to estimate the number of child diarrheal deaths that could be averted for three scenarios where water and sanitation interventions were scaled up [27]. Additionally, LiST was used to create global impact estimates for the *Disease Control Priorities 3rd Edition* publication, determining that scale-up of essential preventative and therapeutic intervention packages to 90% coverage could avert 149,000 maternal deaths, 849,000 stillbirths, 1,498,000 neonatal deaths and 1,515,000 child deaths [28]. A LiST analysis in UNICEF’s *A Promise Renewed 2015* showed that when coverage levels are scaled up to those of the richest in-country wealth quintile, 1.3 million under-five deaths could be averted in a set of 63 priority countries [29].

The value of LiST in these examples, according to respondents, is that it allows MNCH experts to communicate in a way that general audiences can understand. Using LiST, a set of interventions can be translated into the number of “lives saved.” As one of the respondents in our qualitative interviews said:

*“Mainly for us, it is communication. It is the simplest way to communicate results internally and externally is through deaths [sic]. For us, what we have direct measurement of is coverage, through household surveys. That’s our primary measuring stick. For communication, it’s often difficult to communicate what the impact of coverage changes are in terms of health outcomes. That’s what LiST has been most useful for, is the external communication.”*

*-Monitoring and evaluation manager at an NGO*

A related purpose of LiST results, similar to advocacy analyses, is their use in gaining funding for programs. Most donors seek to identify some cost-effectiveness metric. “Lives saved” is a tangible metric that enables donors to understand the impact of their investments. This benefit of LiST was often mentioned in the qualitative interviews:

*“When we are seeking funding from Congress, we can present potential lives saved if we scale certain interventions [and] if more resources are allocated into certain areas.”*

*-Monitoring and evaluation manager at a development and aid agency*

*“You are not going to get anyone to buy in unless you have real data that can encourage them to step up and do something about the issue.”*

*-Program associate at an NGO*

### Capacity building for use of LiST

#### Institutional uptake

Another topic of interest is the way in which LiST is used within organizations. Today, many tools are available to organizations to aid policy-making decisions, but not all tools are equally practical. Organizations need tools that are accessible, simple to use and have the ability to produce outputs that are easily and reliably interpreted.

From our qualitative survey data it seems that, for some organizations, LiST *has* been a practical tool that staff members have been able to learn to use independently and without direct assistance from the LiST team. Organizations have staff members trained to use the tool, particularly in their monitoring and evaluation teams, as noted by one respondent:

*“We are building a team of colleagues at [our organization] who can use the tool … we have [approximately] five people on the team”*

*-Senior director of program monitoring and evaluation at an NGO*

By contrast, other groups recruit outside experts who are trained in LiST to conduct analyses. For instance, an interviewee from World Vision mentioned that the NGO recruits fellows from the Johns Hopkins School of Public Health who are trained to use LiST. Other organizations seek technical assistance directly from the LiST team at Johns Hopkins University. Notably, USAID has contracted members of the LiST team to create models for their *Acting on the Call* reports [30–32]*.*

#### Training, information, and support

Part of using LiST, as with any software, is understanding where to go for technical support. Our quantitative survey showed that around one-third of users get their information about how to use LiST directly from LiST team members, which may indicate that available resources are poorly advertised. Additionally, users reported that being in contact with the LiST team was helpful both for technical troubleshooting and in terms of interpreting results. For instance, one user mentioned:

*“We’ve been able to get in touch with [LiST team members] and we’ve really benefited from having connections to troubleshoot with people in charge.”*

*-Consultant for an international organization*

Though the LiST team is open to helping users, those who do not know the team should be able to get the assistance they need. A lack of awareness about training resources appears to be a potential bottleneck to using LiST. Though our in-person trainings provide users with support and resources, those who cannot attend face barriers to learning the tool. A majority of our quantitative survey respondents (over 50%) reported that they prefer online webinars for training, which would increase access to our global users.

### Linkages with LiST and other tools

As previously outlined, LiST is primarily used as a standalone model. However, LiST also contributes estimates of impact for other models. The OneHealth Tool, the Impact Calculator and EQUIST all use LiST to estimate measures of maternal, newborn and child health that LiST does not produce.

#### OneHealth Tool

The OneHealth Tool, also part of the Spectrum software package, uses LiST in order to provide users with information to aid strategic planning in the health sector. The tool combines other Spectrum modules so that human resources, coverage and costing are all included as inputs. The cause of death structure, effectiveness values and coverage data from LiST are fed into OneHealth, where the user can update or change data [33].

#### Impact Calculator

Population Services International (PSI) uses LiST to generate outputs for its Impact Calculator tool [34]. First, PSI creates projections for a country of interest. A chosen intervention such as use of oral rehydration solution is scaled up from the default baseline to 100%. The result from LiST, deaths averted, is then used to create a “deaths averted coefficient” by dividing the number of deaths averted by the number of interventions needed to reach 100% coverage, which is determined by PSI. Then, the years of life lost (YLL) due to premature death and the years lived with disability (YLD) is estimated based on the *Global Burden of Disease*. Then, the YLL is multiplied by the “deaths averted coefficient” to get the YLL averted per intervention. Next, the YLD averted per intervention is calculated and added to the YLL averted per intervention to get the disability adjusted life years (DALYs) averted per intervention.

#### EQUIST

UNICEF’s EQUIST tool is a web-based platform that aims to identify ways to reduce mortality by closing the equity gap. The model incorporates LiST as one of the components used to weigh various policy options and compare estimated impact. EQUIST uses LiST outcomes to estimate impact on stunting and mortality. Specifically, the total number of deaths, deaths averted by cause and deaths averted by intervention are inputted into the EQUIST tool. These outputs are then used to calculate Early Childhood Disability Adjusted Life Years (ECDALYs) [35].

## Discussion

The Lives Saved Tool has played an important role in modeling the impact of health interventions aimed to improve maternal, newborn and child health. As outlined in this paper, LiST has been used for evaluation, strategic planning and advocacy purposes. Some users have incorporated LiST into their organization’s core operations, while others use LiST for smaller or one-off projects. Regardless, users have noted that LiST is a useful tool with a strong evidence base.

Over the years, LiST has been updated and modified to incorporate users’ needs. The international community’s shift toward addressing health outcomes in the poorest wealth quintiles sparked the development of the Equity Tool within LiST [36]. The Missed Opportunities Tool gives users the ability to quickly identify interventions that will have the biggest impact on MNCH [37]. Additionally, the Subnational Wizard was developed out of the growing need to develop health plans at the state and district levels. Furthermore, the LiST team now holds training webinars and hosts a forum to increase access to training resources. As with any model, LiST will continue to be improved, but not without feedback from our users. Another paper featured in this supplement outlines what users would like to see from LiST in the future [38].

The continuation of LiST is a community effort. The data from household surveys, effectiveness values and cause of death structure that LiST uses to estimate mortality are sourced from outside groups. Efforts to improve coverage data in LMICs can ensure more high-quality data in the future [39]. As the global community seeks to further improve maternal, newborn and child health, we anticipate LiST to be even more useful in the years to come.

### Limitations

This study has several limitations. First, the use of the LiST email mailing list for sampling introduces a bias, as those who regularly interact with the LiST team are more likely to know about the mailing list and receive messages. Additionally, the sampling of the qualitative and quantitative surveys may not be representative of all LiST users. The majority of quantitative survey respondents were from academia and NGOs. Though the literature search included grey literature, many LiST analyses may be unpublished or in private documents. Our qualitative survey results indicated that some organizations use LiST internally and do not make the results available publically. Lastly, the literature search only consisted of articles in English, and important analyses done in other languages were not included in this study.

## Conclusions

The Lives Saved Tool has made important contributions to modeling maternal, newborn and child health through evaluation, strategic planning and advocacy. In the future, LiST should continue to be a useful model for academics, ministries of health, NGOs and intra-governmental groups if the process remains in place to update the model with new scientific evidence and expand the model to meet the needs of its users.

## Additional files

Additional file 1: LiST User Questionnaire. (PDF 139 kb)

Additional file 2: Qualitative survey. (PDF 33 kb)

## Abbreviations

AIM AIDS: Impact Module
DALY: Disability-adjusted life year
ECDALY: Early-childhood disability-adjusted life year
iCCM: Integrated community case management
LiST: Lives Saved Tool
LMIC: Low-to-middle-income country
NGO: Nongovernmental organization
PSI: Population Services International
YDL: Years lived with disability
YLL: Years of life lost
