# Supplementary material for: How is the Lives Saved Tool (LiST) used in the global health community? Results of a mixed-methods LiST user study
Source: BMC Public Health. 2017 Nov 7;17(Suppl 4):773. doi: 10.1186/s12889-017-4750-5 (PMC5688436; doi:10.1186/s12889-017-4750-5)
Supplement: Supplementary file 1 — LiST User Questionnaire. (PDF 139 kb) [file 12889_2017_4750_MOESM1_ESM.pdf]

User Survey

* Required

Name *

Job Title

Email address *

Organization

General Information

1. How often do you use LiST for analysis? *

- Weekly
- Monthly
- Quarterly
- Annually
- Less than once a year
- I have never used LiST for analysis

2. When was the last time you used LiST for analysis? *

- In the past month
- In the past 3 months
- In the past 6 months
- In the past year
- More than one year ago

3. How many others in your organization use (or know how to use) LiST? *

- None
- 1-5
- 5-10
- 10-20

- More than 20

4. Where do you get information about how to use LiST? *

- Johns Hopkins website
- Lives Saved Tool website
- Avenir Health website
- LiST Help menu
- Communication with members of Baltimore LiST team
- Communication with Avenir staff
- I have not looked for information about how to use LiST
- Spectrum and One Health Tool Support Site
- Other:

5. For which of the following purposes do you use LiST? *

Select all that apply

- Program monitoring/evaluation
- Setting policy targets
- Planning
- Advocacy
- Research
- Modelling changes in mortality from coverage results
- Determining coverage increases necessary to achieve target mortality reductions
- As part of a OneHealth application
- None
- Other:

6. For which of the following applications do you use LiST? *

Select all that apply

- Maternal health
- Child health
- Nutrition
- Costing
- Subnational projections

- ☐ None
- ☐ Other: ____

Spectrum Tools

7. How often do you use each of the following Spectrum modules? *

	Often	Occasionally	Never
DemProj	○	○	○
AIM	○	○	○
FamPlan	○	○	○
OneHealth	○	○	○
LiST	○	○	○

8. Do you currently use the Missed Opportunities feature of LiST? *

Note: Missed Opportunities is a feature of the software that projects the impact of each intervention scaled up individually to 90% to quickly assess the most impactful interventions in a given country setting.

- ○ Yes
- ○ No
- ○ No, but now that I know what it does, I'd be interested

9. Do you currently use the Easy LiST feature of LiST? *

Note: Easy List is a feature of the software that lets you calculate results with fewer input parameters.

- ○ Yes
- ○ No
- ○ No, but now that I know what it does, I'd be interested

10. Which of the following results do you use in LiST? *

Select all that apply

- ☐ Total deaths
- ☐ Deaths prevented relative to impact year
- ☐ Cause specific deaths
- ☐ Deaths prevented by cause
- ☐ Deaths prevented by intervention
- ☐ Deaths prevented by intervention by cause

- Mortality rates
- Prevalence of stunting
- Prevalence of wasting
- Breastfeeding prevalence
- Incidence and etiology
- Costs
- None
- Other:

11. Do you use the LiST generated tables and pie charts to communicate results? *

- Yes
- No. I export my results to Excel to create my own tables and charts
- No. I do not use tables and charts to communicate my results

Software Development

12. Which of the following would be useful to you in understanding or communicating your results?

Select all that apply

- I would like to see more visually appealing graphics
- I would like to see more interactive tables and charts
- I would like to be able to export to Powerpoint for chart/table creation
- I do not need more help in understanding or communicating LiST results
- Other:

13. Which of the following applications would you be interested in seeing added to LiST's current capabilities? *

- Equity analysis
- Confidence intervals around number of lives saved
- Expanded costing features
- Analysis of impediments to scale up of coverage
- Expanded capacity to adjust structure or add interventions
- None
- Other:

14. Currently, LiST is a computer-based software that you need to download (requires internet connection), and then run on your computer (does not require internet connection). Would you use a version of the software that had slightly reduced functionality if it could be used online? *

For example, the online version might have fewer options for adjusting Demproj population and fertility parameters; fewer options for specifying HIV inputs and results; fewer options for customizing contraceptive method mix.

- Yes
- No
- Don't know

15. Would you be interested in participating in an on-going online forum where LiST users could ask questions and share experiences about working with the software? *

- Yes
- No

User preferences

16. Please select your preference for notifications about software updates *

- Email
- Website
- Twitter or other social media
- I do not wish to be notified of software updates
- Other: ____

17. Please select your preferences for LiST training workshops *

Select all that apply

- In-person (Baltimore/DC) - 1 day overview workshop
- In person (Baltimore.DC) - 3 day advanced workshop
- Live webinar
- Website - how-to guides
- Website - slides from previous trainings
- Website - videos
- I'm not interested in LiST training workshops
- Other: ____

18. We are forming a 'super-user' focus group which will meet periodically (remotely or in-person) to provide feedback to help developers make improvements in the software. Are you interested in participating? *

Note: If you answer yes, we will contact you via email to follow up.

- Yes
- No

19. Please use the space below to share any information you think would help our developers make the software better meet your specific needs.

Submit

Never submit passwords through Google Forms.

Powered by
