# Supplementary material for: How is the Lives Saved Tool (LiST) used in the global health community? Results of a mixed-methods LiST user study
Source: BMC Public Health. 2017 Nov 7;17(Suppl 4):773. doi: 10.1186/s12889-017-4750-5 (PMC5688436; doi:10.1186/s12889-017-4750-5)
Supplement: Supplementary file 2 — Qualitative survey. (PDF 33 kb) [file 12889_2017_4750_MOESM2_ESM.pdf]

Respondent Code:

Date and time of interview:

General observations:

Summary of key points:

Interview Procedure:

1. Explain the purpose and nature of what we are doing.

We are meeting with you today because we are interested in learning more about how you use the Lives Saved Tool in your public health work, and to identify ways that LiST could be more useful to you. Our aim is to understand how LiST users use this tool in decision-making for maternal and child health.

We will be asking you about how you use LiST in your research/work, to walk us through how you typically use LiST, problems you may have encountered with LiST and your suggestions of areas to work on/improve in LiST.

We expect the interview will take around 30 minutes.

2. Consent for interview and recording (just for notes not for transcription)

I would like to confirm that we have your consent to participate in this study. Additionally, with your consent, I would like to record this interview to assist with note-taking purposes. All information collected in this interview will be kept anonymous, and quotations used in potential publication/reporting will have all personal identifiers removed. Finally, we can stop the interview at any time, and you may opt not to answer a question.

If you have any concerns, please contact Tim Roberton, the PI of the study.

May we begin?

3. General info on respondent

a. What is the name of the organization or university where you are working?

b. What is your role in the organization (i.e. title)?

c. What research do you primarily work on?

4. Information about use of LiST

a. First let's talk about how you generally use LiST. What projects (purposes?) do you primarily use LiST for?

b. How do you use the data generated by LiST?

i. Do you use the data for program planning or to calculate the effect of programs conducted (prospectively or retrospectively)?

c. Can you please picture a specific project where you used LiST, and let's walk through the project step-by-step.

i. Which modules did you use?

ii. Where did you get your data from: did you use default data or input your own data?

iii. How many countries were you pulling data for?

iv. How many indicators were you looking at?

v. How did you use the data generated (export to Excel, copy and paste graphs/tables)?

vi. What was the final output of your research: a paper, publication, external communications?

5. Do others in your organization use LiST?

a. If yes, what projects do they use LiST for?

6. Utility in terms of public health / Strengths / Weaknesses

Now I want to talk more about high-level… the bigger picture…

a. How does using LiST help you in achieving you or your organization's goals?

b. Have you used other similar modeling software?

i. If yes, how does LiST compare?

ii. Why do you prefer to use LiST?

c. Where do you see the future of modelling? Program planning?

d. What new tools for modelling do policy makers need in the next five years?

i. How could LiST fit in, in the future or not?

ii. How could LiST be modified/improved to meet these needs?

7. Issues with Software

a. Have you experienced problems with LiST's functionality?

i. Can you please describe the problem(s)?

b. What are your thoughts on the user interface?

i. What could be improved?

c. How often do you use the graphs/tables generated by LiST?

i. Is the ability to generate graphs and tables within LiST important to you?

8. Suggestions/feedback

a. Is there anything you would like to see changed in the software? What new functionality/tools would you like to see added?

i. Elaborate on the details

Any other comments about anything...
